# Mental health in immigrant men and women in Australia: the North West Adelaide health study

**DOI:** 10.1186/1471-2458-14-1111

**Published:** 2014-10-28

**Authors:** Melanie Straiton, Janet F Grant, Helen R Winefield, Anne Taylor

**Affiliations:** Division of Mental Health, Norwegian Institute of Public Health, Oslo, Norway; School of Psychology, University of Adelaide, Adelaide, Australia; Population Research & Outcome Studies, School of Medicine, University of Adelaide, Adelaide, Australia

**Keywords:** Immigrant health, Mental health, Health inequalities, Health service use

## Abstract

**Background:**

There is conflicting evidence of the healthy migrant effect with respect to mental health. This study aims to determine if there are differences in mental health and service use between Australian-born and foreign-born individuals living in South Australia and to consider the differing role of socio-demographic characteristics for Australian-born and foreign-born men and women.

**Methods:**

Data from the North West Adelaide Health study was used to compare foreign-born men and women from English and non-English speaking backgrounds with Australian born men and women on four measures of mental health and service use. A series of logistic regression analyses were conducted.

**Results:**

There were no differences between Australian-born and foreign-born individuals from English-speaking backgrounds on any measures. Men from non-English speaking backgrounds had higher odds of depression. Employment and general health were important protectors of mental health for both Australian and foreign-born individuals, while being married was protective for foreign-born men only. Income was generally inversely related to mental health among Australians but the relationship was weaker and less consistent for those born abroad.

**Conclusions:**

Men from non-English speaking backgrounds men may be at increased risk of mental health problems but do not have higher levels of treatment. Help-seeking may need to be encouraged among this group, particularly among unmarried, unemployed men from non-English speaking backgrounds.

## Background

In many countries, there has been an increased focus on equal access to health care services across all social groups, including immigrants [1]. Research suggests that foreign born individuals (FBs) may use mental health services less than the rest of the population [2]. While it is recognised that many experience barriers to accessing care [3], there is also the suggestion that FBs experience better mental health [4]. This is known as the healthy migrant effect, where those who (voluntarily) move are assumed to be healthier and more resourceful than those who remain in their home country, upon migration. It is now widely acknowledged that this advantage deteriorates over time [5].

There is conflicting evidence for the healthy migrant effect with respect to mental health [4, 6]. This may be somewhat dependent on the FB population of interest, the host country and the way in which mental health is defined. The current study investigates differences in mental health service use and in mental health between Australian-born (AB) and FB individuals living in South Australia. It also looks at differences and similarities in the socio-demographic factors associated with mental health and service use for AB and FB men and women.

In Australia, around 27.7% of the population are estimated to be FB [7]. The largest groups are from the United Kingdom and New Zealand, but the majority come from non-English speaking backgrounds (NESBs). Recent years have seen a substantial increase in migrants from Asia; with nine of the top ten groups coming from Asian countries from 2007 onwards [8]. In earlier years, European migrants predominated. The largest NESBs groups are from India and China. Despite the large FB population, and the cultural, linguistic and religious diversity within Australia, there is limited research concerning the prevalence of mental health problems and service use in FB populations [2].

### Disparities in mental health and service use in Australia

The Australian healthcare system is universal, aiming for equal access for everyone through the publically-funded Medicare system. Medicare heavily subsidises the cost of health care. FBs who have permission to work in Australia, who have citizenship or a permanent visa, or who are married to an Australian citizen are entitled to enrol in the system [9]. Despite this equity principle, in terms of mental health service use, NESB-FBs appear to have fewer admissions to inpatient wards and are less likely to attend an outpatient clinic compared with ABs [2, 10, 11]. Rates of involuntary admissions however, are higher [12], suggesting that NESB-FBs are more likely to be in a crisis situation when they access care. In support of this, NESB-FBs who are in treatment are more likely to have psychosis but less likely to have less severe mental health problems compared with the general population [2]. Little is known about mental health service use among foreign-born individuals with English-speaking backgrounds (ESB-FBs). Further, the majority of mental health problems are managed at the primary care level and often treated with psychotropic drugs [13]. Rates of psychotropic drug use vary among FBs; some groups have higher rates than ABs and others have lower rates [11].

For mental health disorders, some studies suggest that the Australian-born population have higher rates than foreign-borns, especially NESB-FBs [8, 14]. Other studies find no differences between ABs and ESB-FBs but that NESB-FBs actually report poorer mental health [15, 16]. The conflicting findings may be somewhat due to the measures used; the former two studies focused on diagnosable disorders while the latter studies used self-administered screening instruments for distress.

In the overall Australian population, women report more psychological distress and are more likely to visit a health professional for a mental health problem [17, 18] than men. Despite these known differences, gender has rarely been treated as more than just a risk factor in the above studies, which may disguise important inequalities between FB and AB populations.

### What explains the disparities in mental health?

Differences in the socio-demographic profiles of FBs and ABs may account for much of the differences in mental health and health service use between these groups [19, 20]. However, socio-demographic variables may impact on mental health in different ways for FB and AB individuals. Low socioeconomic status (SES) for instance, has been long linked to poorer mental health in the general population [21]. Yet there is the paradox among Mexican immigrants in the USA, who appear to have better mental health despite having on average, poorer SES than the general population [22]. The relationship between SES and health service use is not clear cut among foreign-born populations [23, 24].

Further, SES can be measured in different ways such as through income level, employment or education. Different ways may relate to mental health differently [25]. For instance, in terms of access to health care services, higher levels of education may allow greater understanding and faster navigation of a health system, while higher income levels may afford a healthier lifestyle and more opportunities, which can protect against mental health problems. The use of multiple measures of SES is therefore recommended [26].

### Current study

The current study aims to assess differences in AB and FB men and women’s mental health using four different measures: diagnosed mental health problems, current depression, mental health service use and use of medication. We hypothesise that NESBS-FBs will have higher rates of current depression than ABs but lower rates of mental health service use, medication and diagnoses. Since studies mostly suggest strong similarities between ABs and FBs with an English-speaking background, we do not expect significant differences between ESB-FBs and ABs. Further, socio-demographic variables may differentially relate to different measures of mental health for FBs than for ABs, as well as for men and women. We therefore compare the association between socio-demographic factors and each measure of mental health for AB and FBs, stratified by gender, to identify variables common to all men and women and those that are unique to foreign-borns.

## Methods

The North West Adelaide Health Study (NWAHS) is a representative biomedical cohort study with a focus on chronic disease and is a collaboration between The University of Adelaide, the South Australian Department for Health and Ageing, the University of South Australia, The Queen Elizabeth and Lyell McEwin Hospitals, and South Australia Pathology (Institute of Medical & Veterinary Science). Ethical approval for this study was sought and granted from the North Western Adelaide Health Service – Ethics of Human Research Committee.

In the initial stage of NWAHS, conducted between 1999 and 2003, all households with a telephone in the north-western area of Adelaide, South Australia, were eligible for selection. Within each contacted household, the person who last had their birthday and who was aged 18+ years was invited to participate. No substitutions for refusal were permitted. Of the original 8213 people who were contacted, 4056 participated, giving an overall response rate of 49.4%. The second wave, conducted between 2004 and 2006 was the first to include several measures of mental health. Data collection for both stages included a telephone interview using Computer Assisted Telephone Interview technology, a self-completed questionnaire and a clinical assessment. The current study uses data from the Stage 2 telephone interview and the questionnaire which includes a total of 3259 participants (80% of initial sample). Socio-demographic factors may have a different relationship with mental health post and pre-retirement age. Thus, in this study, we focus only on adults who were under 65 years at time of data collection (n = 2609). We were missing information on country of birth for 4 individuals. Analyses were therefore based on a total of 2605 individuals. All data collection was conducted in English. Full details of the NWAHS study and data collection procedures have been extensively described elsewhere [27, 28].

### Measures

Four measures of mental health were used as dichotomous dependent variables

- Diagnosis: Participants were asked if they had, in the last 12 months, been told by a doctor that they have any of the following conditions: anxiety, depression, stress related problem, any other mental health problem.
- Current depression: This was measured using the Centre for Epidemiologic Studies for Depression Scale (CESD), a 20-item questionnaire measuring the frequency of depressive symptoms over the last week (rarely or none of the time, some or a little of the time, occasionally or a moderate amount of time, most or all of the time). Total scores range from 0–60, with scores of 16+ indicating some level of depression.
- Medication: Participants were asked if they were currently taking any medication for a mental health problem.
- Consultation: Participants indicated if they had seen a psychologist or a psychiatrist in South Australia in the last 12 months.

The first two variables are collectively referred to as mental health status, while the latter two are referred to as treatment.

The independent variable of interest was birth category. In line with the Australian Bureau of Statistics [29], those born in Australia were categorised as Australian-born, while those born in UK, Ireland, New Zealand, USA, Canada or South Africa were categorised as foreign-born with English-speaking background (ESB-FB). All others were categorised as foreign-born with non-English-speaking background (NESB-FB). As such, the NESB-FB group is far more heterogeneous than the other two groups, coming from over 40 different countries. Around 76% were born in European countries.

Socio-demographic covariates included: age group (20–34, 35–44, 45–54, 55–64); marital status (married/living with partner, never married or previously married); education level (secondary, certificate/diploma/trade or Bachelor degree or higher); gross annual household income (less than $20,000, $20,001-40,000, $40001-60,000, $60,001+), employment status (employed, not part of the workforce (retired/home duties/ student) or unemployed/other/not-reported). ‘Other’ includes mostly disability benefit and long-term sickness. For FB participants, we also included length of stay (20+ years or less than 20 years), estimated based on respondents’ reported year of arrival, assuming the year of data collection was 2005 for all participants. Our final covariate was a self-reported measure of general health (excellent/very good/good or fair/poor).

### Statistical analysis

Chi-square analyses and one way ANOVAs were conducted to assess for differences in socio-demographics between the AB, ESB-FB and NESB-FB groups. Logistic regression was used to assess for differences between the AB group and the ESB-FB and NESB-FB groups on each of the four dependent variables, while 1) adjusting for age-group, 2) additionally adjusting for marital status, education, annual household income, employment status and living situation and 3) additionally adjusting for general self-reported health. Analyses were stratified by gender.

To determine associations between socio-demographic factors and each of the dependent variables, univariate logistic regression analyses were conducted separately for AB and FB men and women. Variables with an overall *p* value of 0.25 or less (based on the Wald statistic) were entered into a multivariate analysis [30]. Non-significant variables (0.05 and above) were removed, one by one, until all variables remaining in the model were significant. To preserve power, both ESB-FB and NESB-FB groups were grouped together, but in the final models we also checked for interactions between birth group and each variable to determine whether any variables had a different association with the dependent variable for ESB-FBs and NESB-FBs.

Data were analysed using SPSS. Weightings by age group, sex, region and the probability of selection in the household according to the 2004 Estimated Resident Population were applied to all analyses [31]. This ensured that the sample was representative of the population in the northern and western suburbs of Adelaide.

## Results

Table 1 displays the demographic profiles of the sample, by birth category. There were overall differences between the three groups in terms of the proportion of individuals who were married (lowest among AB), had higher education (highest among AB), had a household annual income of less than $20,000 (highest among NESB-FB), were currently employed (highest among AB) and had only fair or poor general health (highest among NESB-FB). AB also had a lower mean age than both groups of FBs. A lower proportion of NESB-FB than ESB-FBs had been in Australia less than 20 years.

**Table 1:** **Socio-demographic characteristics of the sample**

	Australian-born	Foreign-born (English speaking background)	Foreign-born (non-English speaking background)
	N = 1895	N = 417	N = 292
% women	50.6	48.5	43.6
% married*	66.6	76.3	75.4
% with higher education*	22.7	14.2	15.7
% income less than $20,000*	11.6	10.8	16.1
% currently employed*	77.5	73.7	68.2
% living alone	9.5	8.5	5.5
% length of stay <20 years*	-	16.4	25.4
% reporting fair/poor health*	11.2	10.6	21.2
Mean age in years (sd)*	39.0 (11.7)	47.3 (10.5)	46.3 (11.0)

*p < 0.05.

Overall, 16.3% of the sample reported having a diagnosis in the past 12 months and 14.3% scored higher than 16+ on the CESD, indicating some level of current depression. In terms of treatment, 9.0% of the sample was on medication and 5.6% had visited a psychologist or psychiatrist in the past 12 months. Table 2 shows these percentages by birth category, stratified by gender. Among men, we found higher odds of a diagnosis and of current depression among NESB-FBs compared with ABs. Accounting for self-reported general health rendered this difference non-significant for a diagnosis but not for current depression. When adjusting for age, there were no significant differences between AB and ESB-FB men and thus, further adjustments were not made. For women, there were no significant differences between AB and either of the FB groups.

**Table 2:** **Percentages, odds ratios (OR) and 95% confidence intervals (CI) on mental health measures by birth category and gender**

Men	Australian-born (AB)	Foreign-born: English-speaking (ESB-FB)	Foreign-born: non-English-speaking (NESB-FB)	AB vs. ESB-FB	AB vs. NESB-FB	AB vs. ESB-FB	AB vs. NESB- FB	AB vs. ESB- FB	AB vs. NESB- FB
				OR (95% CI) ^1^	OR (95% CI) ^1^	OR (95% CI) ^2^	OR (95% CI) ^2^	OR (95% CI) ^3^	OR (95% CI) ^3^
Diagnosis	12.9%	14.1%	22.0%	1.08 (0.68-1.73)	1.86 (1.17-2.95)*	1.05 (0.65-1.71)	1.73 (1.08-2.79)*	1.02 (0.96-2.59)	1.58 (0.96-2.59)
Current depression	10.1%	9.2%	19.7%	0.92 (0.53-1.60)	2.22 (1.37-3.58)*	0.91 (0.51-1.62)	2.02 (1.22-3.36)*	0.88 (0.49-1.59)	1.84 (1.09-3.10)*
Medication	6.7%	8.1%	6.1%	0.91 (0.50-1.66)	0.77 (0.36-1.64)	-	-	-	-
Consultation	4.9%	6.5%	6.8%	1.22 (0.63-2.37)	1.31 (0.62-2.77)	-	-	-	-
Women									
Diagnosis	18.2%	22.8%	17.8%	1.32 (0.88-1.99)	0.94 (0.54-1.65)	-	-	-	-
Current depression	18.1%	13.5%	20.2%	0.75 (0.47-1.21)	1.26 (0.74-2.15)	-	-	-	-
Medication	11.6%	12.7%	5.8%	1.16 (0.67-1.92)	0.45 (0.19-1.10)	-	-	-	-
Consultation	6.3%	5.2%	4.0%	0.83 (0.39-1.76)	0.70 (0.25-1.95)	-	-	-	-

^1^adjusted for age, ^2^additionally adjusted for marital status, education level, annual household income, employment status and living situation, ^3^additionally adjusted for general health. *p < 0.05; − results not reported due to lack of significant finding in earlier stage of analysis.

Table 3 displays the association between socio-demographic variables and each dependent variable, separately for AB and FB men. Employment and general self-reported health were related to most measures of mental health across both groups. Having fair or poor general health was associated with higher odds of poor mental health status and of treatment compared with those in good health. Being unemployed/other was also associated with higher odds compared with those who were employed. Additionally, for AB men, those not in the workforce had higher odds on all measures. Marriage and living with others were associated with mental health status and treatment for FB men. Education level (having a trade/certificate/diploma) was related to treatment for both AB and FB men. Age showed no consistent relationship.

**Table 3:** **Odds ratios (OR) and 95% confidence intervals (CI) for variables tested for Australian-born and foreign-born men**

	Australian-born men				Foreign-born men			
	Diagnosis	Current depression	Medication	Consultation	Diagnosis	Current depression	Medication	Consultation
Age group								
20-34	0.66 (0.39-1.13)	1.17 (0.60-2.30)	0.27 (0.12-0.61)*	0.30 (0.13-0.67)*	1.47 (0.71-3.07)	1.20 (0.50-2.88)	0.22 (0.05-0.99)*	1.47 (0.51-4.28)
35-44	0.59 (0.33-1.06)	0.95 (0.46-1.98)	0.73 (0.35-1.52)	0.48 (0.22-1.09)	0.33 (0.12-0.90)*	0.87 (0.34-2.21)	0.34 (0.09-1.27)	0.87 (0.26-2.90)
45-54	0.64 (0.34-1.21)	1.27 (0.60-2.68)	1.13 (0.55-2.36)	0.57 (0.24-1.32)	0.53 (0.24-1.19)	0.77 (0.32-1.85)	0.58 (0.21-1.57)	0.15 (0.02-1.05)
55-64	1.00	1.00	1.00~	1.00~	1.00~	1.00	1.00~	1.00~
Marital status								
Married	1.00	1.00~	1.00~	1.00	1.00~	1.00~	1.00~	1.00~
Never married	1.00 (0.64-1.54)	1.50 (0.94-2.38)	0.82 (0.44-1.56)	0.93 (0.47-1.87)	5.51 (2.71-11.20)*	3.49 (1.65-7.39)*	2.89 (0.99-8.43)	3.87 (1.31-11.47)*
Previously married	1.52 (0.77-2.95)	2.41 (1.23-4.72)*	3.22 (1.60-6.50)*	2.03 (0.83-4.99)	3.91 (1.73-8.86)*	1.59 (0.59-4.31)	4.94 (1.75-13.94)*	5.85 1.96-17.46)*
Education								
Secondary	1.00	1.00~	1.00~	1.00~	1.00~	1.00~	1.00~	1.00~
Trade/cert/diploma	1.01 (0.65-1.56)	0.62 (0.39-0.99)*	0.36 (0.20-0.63)*	0.46 (0.22-0.94)*	0.46 (0.24-0.88)*	0.61 (0.31-1.22)	0.38 (0.14-0.99)*	0.18 (0.06-0.56)*
Bachelor or higher	1.30 (0.79-2.12)	0.62 (0.35-1.10)	0.26 (0.11-0.58)*	1.18 (0.60-2.32)	0.45 (0.18-1.11)	0.44 (0.16-1.26)	0.57 (0.17-1.91)	0.44 (0.13-1.52)
Income								
Up to $20,000	2.36 (1.27-4.37)*	3.30 (1.68-6.50)*	6.67 (3.23-13.83)*	8.55 (3.12-23.44)*	2.20 (0.97-5.02)	3.84 (1.50-9.79)*	16.23 (4.35-60.59)*	2.30 (0.59-8.94)
$20,001-40,000	1.30 (0.78-2.18)	2.59 (1.51-4.44)*	2.38 (1.19-4.78)*	3.86 (1.48-10.05)*	1.17 (0.55-2.47)	2.97 (1.29-6.80)*	2.54 (0.57-11.35)	3.51 (1.19-10.35)*
$40,001-60,000	1.15 (0.72-1.84)	1.30 (0.75-2.28)	1.20 (0.57-2.52)	4.60 (1.92-11.04)*	0.34 (0.13-0.89)*	0.62 (0.21-1.86)	1.99 (0.44-8.90)	0.47 (0.09-2.53)
Over $60,000	1.00~	1.00~	1.00~	1.00~	1.00~	1.00~	1.00~	1.00~
Employment								
Employed	1.00~	1.00~	1.00~	1.00~	1.00~	1.00~	1.00~	1.00~
Not in workforce	1.92 (1.16-3.43)*	2.30 (1.30-4.08)*	2.13 (1.03-4.41)*	7.73 (3.99-14.95)*	1.40 (0.52-3.77)	1.66 (0.57-4.88)	4.11 (1.11-15.15)*	0.69 (0.09-5.50)
Unemployed/other	2.77 (1.33-5.77)*	2.37 (1.03-5.45)*	7.92 (3.77-16.64)*	7.85 (3.21-19.23)*	2.20 (0.99-4.84)	3.48 (1.55-7.80)*	10.11 (3.81-26.80)*	4.23 (1.57-11.43)*
Living situation								
Lives with others	1.00~	1.00~	1.00~	1.00	1.00~	1.00	1.00~	1.00~
Lives alone	1.41 (0.81-2.45)	1.51 (0.83-2.75)	1.60 (0.80-3.22)	1.24 (0.52-2.95)	3.26 (1.40-7.63)*	1.21 (0.41-3.62)	3.89 (1.34-11.31)*	6.49 (2.32-18.06)*
General health								
Good/very good/excellent	1.00~	1.00~	1.00~	1.00~	1.00~	1.00~	1.00~	1.00~
Fair/poor	3.62 (2.30-5.69)*	3.76 (2.31-6.11)*	4.28 (2.46-7.46)*	4.23 (2.42-8.45)*	7.90 (4.05-15.38)*	6.16 (3.05-12.44)*	4.35 (1.78-10.64)*	10.39 (4.07-26.52)*
Length of stay	-	-	-	-				
< 20 years	-	-	-	-	0.65 (0.30-1.41)	0.50 (0.20-1.25)	0.16 (0.02-1.16)	2.36 (0.94-5.94)
≥20 years	-	-	-	-	1.00	1.00~	1.00~	1.00~
Background	-	-	-	-				
English-speaking	-	-	-	-	1.00~	1.00~	1.00	1.00
Non-English speaking	-	-	-	-	1.67 (0.93-3.00)	2.43 (1.26-4.68)*	0.75 (0.31-1.83)	1.00 (0.41-2.44)

*p < 0.05, ~p < 0.25 for overall Wald statistic and variable included in multivariate model; − length of stay/background not relevant for Australian-born.

Associations between socio-demographic variables and each of the dependent variable are displayed in Table 4 for women. Again, being unemployed/other and having poorer health was associated with higher odds on most measures. Due to the small number of FB women aged 20–34, the two youngest age categories were collapsed for this group (20–44, 45–54, 55–64). Compared with AB women aged 55–64, all younger groups had higher odds of a consultation. Previously married AB women also had higher odds on all measures compared with married women but variable was not important for FB women.

**Table 4:** **Odds ratios and 95% confidence intervals for variables tested for Australian-born and foreign-born women**

	Australian-born women				Foreign-born women			
	Diagnosis	Current depression	Medication	Consultation	Diagnosis	Current depression	Medication	Consultation
Age group								
20-34	1.44 (0.83-2.49)	1.39 (0.81-2.39)	1.91 (0.96-3.82)	3.86 (1.04-14.39)*	-	-	-	-
35-44 (20–44)	1.38 (0.77-2.48)	1.44 (0.81-2.55)	1.17 (0.54-2.51)	3.91 (1.01-15.08)*	0.89 (0.39-2.03)	1.29 (0.55-3.02)	0.79 (0.27-2.35)	0.25 (0.03-1.80)
45-54	1.53 (0.83-2.78)	1.19 (0.65-2.18)	1.99 (0.94-4.22)	4.30 (1.10-16.89)*	2.43 (1.18-5.00)*	1.71 (0.76-3.89)	1.66 (0.65-4.23)	1.35 (0.40-4.54)
55-64	1.00	1.00	1.00~	1.00~	1.00~	1.00	1.00	1.00~
Marital status								
Married	1.00~	1.00~	1.00~	1.00~	1.00~	1.00~	1.00~	1.00
Never married	0.97 (0.64-1.45)	1.15 (0.77-1.71)	1.19 (0.74-1.91)	1.34 (0.72-2.51)	0.08 (0.00-1.41)	0.38 (0.07-2.05)	0.20 (0.01-3.70)	-
Previously married	1.83 (1.15-2.89)*	2.26 (1.44-3.55)*	2.25 (1.33-3.80)*	2.50 (1.29-4.86)*	1.59 (0.70-3.61)	1.69 (0.70-4.12)	2.49 (0.94-6.57)	2.29 (0.60-8.75)
Education								
Secondary	1.00~	1.00~	1.00~	1.00	1.00~	1.00~	1.00	1.00
Trade/cert/diploma	0.86 (0.60-1.24)	0.73 (0.51-1.05)	1.29 (0.84-1.97)	1.29 (0.73-2.29)	0.50 (0.26-0.98)*	0.54 (0.26-1.12)	0.50 (0.20-1.24)	1.39 (0.42-4.56)
Bachelor or higher	0.55 (0.35-0.87)*	0.27 (0.16-0.46)*	0.71 (0.42-1.23)	0.87 (0.43-1.74)	0.78 (0.31-1.95)	0.65 (0.23-1.89)	0.75 (0.21-2.64)	0.99 (0.15-6.62)
Income								
Up to $20,000	3.59 (2.32-5.55)*	2.85 (1.85-4.40)	4.47 (2.72-7.35)*	3.55 (1.91-6.61)*	0.96 (0.40-2.31)	2.28 (0.80-6.45)	1.60 (0.58-4.40)	1.29 (0.31-5.32)
$20,001-40,000	1.62 (1.03-2.54)*	1.54 (0.99-2.39)	1.63 (0.94-2.83)	0.72 (0.30-1.72)	0.55 (0.25-1.22)	1.64 (0.64-4.17)	0.17 (0.03-0.84)*	0.46 (0.10-2.17)
$40,001-60,000	0.99 (0.62-1.59)	0.75 (0.46-1.21)	0.80 (0.43-1.49)	0.91 (0.43-1.94)	0.64 (0.28-1.46)	2.03 (0.79-5.23)	0.68 (0.23-1.96)	0.36 (0.06-2.23)
Over $60,000	1.00~	1.00~	1.00~	1.00~	1.00	1.00	1.00~	1.00
Employment								
Employed	1.00~	1.00~	1.00~	1.00~	1.00~	1.00~	1.00~	1.00~
Not in workforce	0.89 (0.60-1.33)	0.89 (0.60-1.32)	0.71 (0.42-1.21)	0.86 (0.43-1.74)	0.75 (0.39-1.46)	1.01 (0.50-2.04)	0.47 (0.17-1.32)	0.34 (0.06-1.92)
Unemployed/other	6.33 (3.61-11.10)*	3.90 (2.23-6.84)*	8.42 (4.75-14.93)*	9.36 (4.86-18.03)*	6.41 (1.65-24.89)*	3.20 (0.81-12.68)	7.23 (1.96-26.71)*	9.01 (1.95-41.66)*
Living situation								
Lives with others	1.00	1.00	1.00	1.00	1.00	1.00	1.00	1.00
Lives alone	1.14 (0.65-2.10)	0.84 (0.46-1.55)	1.06 (0.53-2.10)	1.19 (0.49-2.91)	0.62 (0.16-2.39)	0.90 (0.24-3.44)	1.33 (0.32-5.44)	2.21 (0.40-12.09)
General health								
Good/very good/excellent	1.00~	1.00~	1.00~	1.00~	1.00~	1.00~	1.00~	1.00
Fair/poor	3.76 (2.44-5.79)*	14.04 (8.91-22.12)*	2.18 (1.30-3.66)*	1.99 (1.01-3.94)*	1.70 (0.76-3.79)	5.15 (2.34-11.35)*	2.77 (1.09-7.03)*	1.01 (0.19-5.41)
Length of stay	-	-	-	-				
< 20 years	-	-	-	-	0.38 (0.14-1.01)	0.51 (0.19-1.41)	0.05 (0.00-1.53)	0.27 (0.03-2.89)
≥20 years	-	-	-	-	1.00~	1.00~	1.00~	1.00
Background								
English-speaking					1.00	1.00~	1.00~	1.00
Non-English speaking					0.72 (0.39-1.36)	1.64 (0.85-3.15)	0.40 (0.15-1.03)	0.84 (0.26-2.76)

*p < 0.05, ~p < 0.25 for overall Wald statistic and variable included in multivariate model; age-groups 20–34 and 35–44 combined for foreign-born women; − length of stay/background not relevant for Australian-born.

Table 5 shows the results of the multivariate analyses for all four measures of mental health for Australian-born men and women. Self-reported general health has a strong association with both mental health status and treatment for AB men and with mental health status for AB women. Employment was also an important variable for both men and women; those who were unemployed/other had higher odds of poor mental health status and of treatment compared with those who were employed. Additionally, men who were not in the workforce had higher odds of current depression and of consultation. Marital status was unimportant for AB men but married AB women had higher odds of a diagnosis and of medication than those who had never been married. Compared with women aged 55–64, younger AB women had higher odds of treatment. Age was unimportant for men. Lower income levels were generally associated with higher odds of medication and poor mental health status for both AB men and women.

**Table 5:** **Odds ratios and 95% confidence intervals for final multivariate model for Australian-born men and women**

	Australian-born men				Australian-born women			
	Diagnosis	Current depression	Medication	Consultation	Diagnosis	Current depression	Medication	Consultation
Age group								
20-34							2.90 (1.32-6.36)*	4.32 (1.12-16.62)*
35-44							1.59 (0.68-3.68)	4.81 (1.19-19.38)*
45-54							2.49 (1.09-5.69)*	5.12 (1.24-21.02)*
55-64							1.00	1.00
Marital status								
Married					1.00		1.00	
Never married					0.58 (0.36-0.93)*		0.46 (0.25-0.84)*	
Previously married					0.82 (0.47-1.43)		0.96 (0.50-1.84)	
Education								
Secondary			1.00	1.00		1.00		
Trade/cert/diploma			0.40 (0.22-0.73)*	0.42 (0.19-0.93)*		0.83 (0.55-1.26)		
Bachelor or higher			0.30 (0.12-0.72)*	1.49 (0.70-3.18)		0.40 (0.23-0.71)*		
Income								
Up to $20,000		2.62 (1.30-5.27)*	2.89 (1.08-7.70)*	1.23 (0.35-4.34)	2.73 (1.48-5.05)*		4.39 (2.12-9.09)*	
$20,001-40,000		2.32 (1.34-4.01)*	1.77 (0.84-3.73)	1.75 (0.58-5.25)*	1.55 (0.95-2.53)		1.89 (1.04-3.42)*	
$40,001-60,000		1.24 (0.70-2.18)	1.12 (0.53-2.39)	4.94 (1.99-12.27)*	1.03 (0.64-1.66)		0.87 (0.46-1.64)	
Over $60,000		1.00	1.00	1.00	1.00		1.00	
Employment								
Employed	1.00	1.00	1.00	1.00	1.00	1.00	1.00	1.00
Not in workforce	1.66 (0.95-2.91)	7.20 (3.12-16.59)*	0.93 (0.37-2.30)	7.20 (3.12-16.59)*	0.71 (0.45-1.10)	0.83 (0.53-1.29)	0.53 (0.29-0.95)*	1.05 (0.51-2.16)
Unemployed/other	2.22 (1.02-4.82)*	12.63 (3.76-42.51)*	4.00 (1.44-11.14)*	12.63 (3.76-42.51)*	3.86 (1.92-7.77)*	3.24 (1.72-6.10)*	3.90 (1.86-8.19)*	10.55 (5.38-20.68)*
General health								
Good/very good/excellent	1.00	1.00	1.00	1.00	1.00	1.00		
Fair/poor	3.26 (2.06-5.18)*	3.31 (2.00-5.46)*	3.16 (1.71-5.82)*	3.64 (1.79-7.38)*	3.12 (1.97-4.93)*	12.26 (7.68-19.56)*		

*p < 0.05; table includes only significant variables.

Results of the multivariate analyses for foreign-born men and women are displayed in Table 6. For FB men, being married was protective of poor mental health status and associated with lower odds of medication, while living alone was associated with higher odds of consultation. Poor self-reported health was also associated with higher odds of current depression and consultations. Employed men had lower odds of being on medication compared with the other groups. Education was only related to consultations; men with a trade/certificate or diploma had lower odds of consultation than those with secondary education. Income was not strongly related the mental health measures for FB men; only those with an income of $20,000-40,000 had higher odds of current depression compared with those on incomes over $60,000. Length of stay related to three measures of mental health for FB men, but the relationship was not consistent; FB men who had been in Australia less than 20 years had higher odds of consultation but lower odds of current depression and of being on medication. For FB women, employment status was the only variable significantly associated with consultations and general health was the only variable significantly associated with current depression in the final analyses. Since odd ratios and confidence intervals are already displayed in Table 4, consultations and current depression are omitted from Table 6. FB women aged 45–54 had higher odds of a diagnosis than women aged 55–64. Having a trade/certificate or diploma was associated with lower odds of a diagnosis compared with those with secondary education. Unemployment was associated with higher odds of both a diagnosis and of being on medication. In contrast to other groups, FB women with a household income of $20,000-40,000 had lower odds of being on medication than women with an income over $60,000.

**Table 6:** **Odds ratios and 95% confidence intervals for final multivariate model for foreign-born men and women**

	Foreign-born men				Foreign-born women	
	Diagnosis	Current depression	Medication	Consultation	Diagnosis	Medication
Age group						
20-34				0.45 (0.08-2.53)	-	
35-44 (20–34)				1.34 (0.30-5.84)	1.15 (0.47-2.85)	
45-54				0.12 (0.02-0.94)*	2.60 (1.18-5.74)*	
55-64				1.00	1.00	
Marital status						
Married	1.00	1.00	1.00			
Never married	4.87 (2.25-10.56)*	4.11 (1.59-10.65)*	3.93 (1.13-13.59)*			
Previously married	4.12 (1.70-9.98)*	1.67 (0.56-4.99)	6.47 (1.99-21.00)*			
Education						
Secondary				1.00	1.00	
Trade/cert/diploma				0.22 (0.06-0.83)*	0.40 (0.19-0.83)*	
Bachelor or higher				0.26 (0.05-1.40)	0.59 (0.22-1.60)	
Income						
Up to $20,000		2.30 (0.81-6.53)				2.08 (0.60-7.22)
$20,001-40,000		3.14 (1.20-8.22)*				0.17 (0.03-0.89)*
$40,001-60,000		0.96 (0.30-3.12)				0.74 (0.24-2.27)
Over $60,000		1.00				1.00
Employment						
Employed			1.00		1.00	1.00
Not in workforce			5.96 (1.47-24.07)*		0.64 (0.30-1.36)	0.44 (0.13-1.51)
Unemployed/other			8.50 (2.99-24.13)*		6.06 (1.37-26.77)*	8.21 (1.87-36.20)*
Living situation						
Lives with others				1.00		
Lives alone				9.78 (2.59-36.94)*		
General health						
Good/very good/excellent		1.00		1.00		
Fair/poor		3.55 (1.60-7.88)*		13.57 (4.06-45.29)*		
Length of stay						
< 20 years		0.23 (0.07-0.78)*	0.12 (0.02-0.91)*	4.94 (1.09-22.32)*		
≥20 years		1.00	1.00	1.00		
Background						
English-speaking	1.00	1.00				
Non-English speaking	7.46 (3.67-15.18)*	2.41 (1.14-5.10)*				

*p < 0.05; table includes only significant variables; age-groups 20–34 and 35–44 combined for foreign-born women.

We found no interactions between English-speaking background and any of the variables in the final models for either men or women, suggesting that these variables did not relate to mental health measures in significantly different ways for ESB-FB and NESB-FBs.

## Discussion

This study aimed to investigate differences in mental health and service use between AB and FBs living in South Australia. As hypothesised, there were no differences between ABs and ESB-FBs on any of our measures. While we expected that NESB-FBs might have lower rates of diagnosed disorders, consultations and medication, we hypothesised that rates of current depression would be higher. This was only partially supported; higher rates of depression were observed among NESB-FB men but not among NESB-FB women. Contrary to expectations, diagnoses were also higher among NESB-FB men compared with AB men, although general health partially explained this difference. In line with recent research, we found no evidence of the healthy migrant effect for mental health in this study [15, 16]. The majority of participants in the current study were however, well-established in Australia; only 3% had lived in Australia less than 10 years. Any initial health benefits in the current sample may have deteriorated, though other recent research found no effect among FBs with shorter length of stays either [15].

In contrast to previous studies suggesting that NESB-FBs may utilise mental health services to a lesser extent than ABs [2], we found no differences between ABs and FBs’ consultation rates. Because NESB-FB men were more likely to have poorer mental health, it may suggest that men in this group do not access treatment equally according to need. Men are often reluctant to seek help for mental health problems [32] and foreign-born report a number of barriers to care including language difficulties [3, 33]. NESB-FB men may therefore need to overcome greater hurdles to access care compared with the other groups. Consideration should be given to ways of making mental health care more accessible for foreign-born men in particular.

Due to the secondary nature of this study, we were limited in the explanatory factors we could adjust for and as a result, the reasons why NESB-FB men are at heightened risk of poor mental health compared with ABs are not clear from this study. Previous research however, has linked to poor mental health among FB men in Australia to factors such as discrimination, pre-migration experiences and social support [16, 20]. It should also be noted that the NESB-FB group is very heterogeneous, coming from a range of different countries and cultures. There is likely to be large within group variation but we lacked the power to investigate differences by country or world region.

For women, there were no differences in mental health status or treatment rates, suggesting that FB women have similar levels of access to care as ABs according to need. This finding is encouraging, although may only be applicable to those with longer lengths of stay. Further, all were proficient enough in English to participate in the study; those who may experience the greatest barriers to care and the poorest mental health may have been excluded. Nonetheless, an interpreter service is available through the Australian health care system.

Although not statistically significant, NESB-FB women were less than half as likely to be on medication as AB women. Lower rates of anti-depressants have been found among immigrants compared with natives in other countries [34]. Mental health problems can be perceived differently across cultures, which can lead to varying ideas about the type of treatments, if any, that are appropriate [35]. Some NESB-FB women may prefer to seek other ways of dealing with mental health conditions.

Women typically report more distress than men [18] and our findings for ABs and ESB-FBs confirmed this. However, in this study, foreign-born men from a non-English-speaking background had notably similar rates of depression as their female counterparts and even higher rates of diagnosis. We did not directly investigate these gender differences and so can only speculate as to the reasons for this difference. NESB-FB men in this sample were less likely to be married than NESB-FB women. Further, marriage and living with others were important protective factors for FB men but not FB women. In general, women more often report friends and relatives as sources of support, while men more often report their spouses as their main source [36]. Upon migration, immigrants experience significant social disruption, loss of social network and often, extended family [37]. Support from one’s spouse may therefore become even more paramount. Mental health promotion campaigns which improve immigrant men’s social networks may be beneficial. For all groups, we found that those with fair or poor general health were more likely to report current depression compared with those with better health. General health also related to a mental health diagnosis among AB men and women in the same way. This consistent association is unsurprising as co-morbidity between general health and mental health is well-documented [38, 39]. NESB-FB men’s poorer general health status relative to AB men’s explained some of the difference in diagnosis. Although the causality is not known, it is conceivable that interventions aiming to improve the general health of NESB-FB men could also improve their mental health.

Being unemployed (or ‘other’) also had a strong association with mental health for AB and FB men and women. Unemployment has long been associated with poor mental health [40]. As with all other associations reported, the causal direction cannot be determined due to the cross-sectional nature of the study, though it is thought to be bidirectional [41, 42]. Unemployment is typically found to have more adverse effects on men’s than women’s mental health [42] but associations were strong among both men and women in the current study. However, we also observed that not being in the workforce was associated with higher odds of depression for AB men. This may relate to conventional gender roles: men who are not in employment may experience a loss of status and greater social stigma due to societies’ expectations. Stay-at-home men who otherwise closely adhere to the traditional masculine gender role may be at heighted risk of poor mental health [43].

As well as employment, this study benefited from the inclusion of income and education as measures of SES. Income appeared to be an important variable for ABs but the relationship was weaker and less consistent for FBs. This may be because foreign-borns, particularly those with a non-English speaking background may predominate in low paid jobs and can experience difficulties in obtaining jobs that match their level of qualification [44]. Yet, education was not strongly related to the measures of mental health for FBs either; it was associated only with having diagnosis for FB women and consultations for FB men. These findings confirm the complexity of the relationship between SES and mental health among FBs [22, 23, 25].

In addition to the sampling limitations mentioned above, this study also excluded people without a telephone or who were not listed in the White pages. However, since 97% of households in the study region have telephones, this is not likely to have significantly affected the representativeness [45]. While the attrition rate of around 10% from stage 1 of recruitment to stage 2 of the study may be a concern, since those with poorer mental health may have been more likely to drop out, there was little difference in attrition rates across the three groups (AB, ESB-FBs and NESB-FB). The validity of the comparisons made in this study is therefore unlikely to have been significantly compromised. All variables of interest were self-reported, which may be vulnerable to socially-desirable responding. This may be heighted among FBs, who may worry that reporting poor mental health will affect their residency status. However, most FBs in this sample were already well-established in Australia so this may be less of an issue.

The study benefits from using multiple measures of mental health, which has allowed us to explore differences in AB and FBs’ mental health status and treatment, as well as identify different variables that relate to different measures. Unlike other Australian studies, it also investigates men and women separately. The weightings applied in the study should make the results generalizable to the wider population in urban South Australia.

## Conclusion

In general, the findings are positive in that FBs appear to access mental health services to a similar degree as ABs. NESB-FB men in this study, however, are at increased risk of mental health problems but do not have higher levels of treatment than AB men. Thus, there may be a need to increase the awareness of mental health problems and help-seeking among this group, particularly among unmarried, unemployed NESB-FB men who live alone. These risk factors also suggest the importance of social support in protecting against mental health problems for NESB-FB men in particular.

## Abbreviations

FBs: Foreign born individuals
ABs: Australian-born individuals
NESB: Non-English speaking background
ESB: English speaking background
SES: Socioeconomic status
NWAHS: The North West Adelaide Health Study.
